# Supplementary material for: Quantum coherence and interference of a single moiré exciton in nano-fabricated twisted monolayer semiconductor heterobilayers
Source: Nat Commun. 2024 Jun 8;15:4905. doi: 10.1038/s41467-024-48623-4 (PMC11162440; doi:10.1038/s41467-024-48623-4)
Supplement: Supplementary file 1 — Supporting Information [file 41467_2024_48623_MOESM1_ESM.docx]

**Supplementary Information**

**Quantum coherence and interference of a single moiré exciton in nano-fabricated twisted monolayer semiconductor heterobilayers**

*Haonan Wang^1^, Heejun Kim^1^, Duanfei Dong^1^, Keisuke Shinokita^1^,*

*Kenji Watanabe^2^, Takashi Taniguchi^3^, and Kazunari Matsuda^1^*

^1^Institute of Advanced Energy, Kyoto University, Uji, Kyoto 611-0011, Japan

^2^Research Center for Electronic and Optical Materials, National Institute for Materials Science, 1-1 Namiki, Tsukuba, Ibaraki 305-0044, Japan

^3^Research Center for Materials Nanoarchitectonics, National Institute for Materials Science, 1-1 Namiki, Tsukuba, Ibaraki 305-0044, Japan

**Supplementary Note 1. Measurement of twist angle in twisted MoSe_2_/WSe_2_ heterobilayers**

**Supplementary Figure 1 (a) Optical image of twisted MoSe_2_/WSe_2_ heterobilayers encapsulated by *h*-BNs before nanofabrication.** Solid blue and orange lines correspond to monolayer MoSe_2_ and WSe_2_ regions, respectively. **(b)** Polarization-resolved SHG measurement. The polar plot of the SHG intensity (points) and fitted curve (solid lines) for the monolayer MoSe_2_ (black), monolayer WSe_2_ (green) and MoSe_2_/WSe_2_ heterobilayer (red), show six-fold symmetry. SHG result of WSe_2_ monolayer shows relatively larger experimental errors, which is caused by limited monolayer area after the nanofabrication. SHG measurements were performed in the unetched areas in the heterobilayers.

The stacking angle between the monolayer MoSe_2_ and WSe_2_ in the twisted heterobilayer is obtained using second-harmonic generation (SHG) measurements^1^. The SHG signals are measured using a linearly polarized pulsed laser with a repetition rate of 80 MHz, pulse duration of 100 fs, and center wavelength of 900 nm, in which the laser is focused on the monolayers MoSe_2_ and WSe_2_ and heterobilayer regions. The angle dependence of the SHG signal is measured via rotating the laser polarization with a half-wave plate. Under these conditions, the maximum SHG intensity is observed when the laser polarization is parallel to the zigzag directions of the monolayer transition metal dichalcogenides. In the heterobilayer region, different stacking types lead to in-phase (R-type) or anti-phase (H-type) relationships between the SHG signals of the two layers, thereby, generating strengthened or weakened SHG intensity.

Supplementary Figure 1(b) presents the angle dependence of the SHG signals of monolayer MoSe_2_ (black)_,_ and heterobilayer (red). The polar plots show six-fold symmetry, and the SHG intensity from the heterobilayer region is greatly reduced, which indicates the H-type stacking of the heterobilayer^2^. The evaluated twist angle between monolayer MoSe_2_ and WSe_2_ is measured to be 56.5° ± 0.3°.

**Supplementary Note 2. Estimation of the optically generated exciton density**

The exciton density is estimated under continuous-wave laser excitation measurement. The equation for estimation is as follows:

$\frac{dN}{dt}$ = *g* − $\frac{N}{\tau_{nrad}}$ − $\frac{N}{\tau_{rad}}$, (S1)

*g* = $\frac{\left( 1-R \right)\alpha dP}{\hbar\omega}$*,* (S2)

where *g* represents the generation rate of moiré exciton, and *N* is the density of moiré exciton. *τ*_rad_ and *τ_n_*_rad_ are the radiative and nonradiative lifetime, respectively. *d* (1.4 nm) is the thickness of the heterobilayer. *P* is the excitation power density. $\hbar\omega$ (2.38 eV), is the excitation photon energy determined by the laser. *R* (0.4) is the reflectivity and *α* (=6 × 10^5^ cm^−1^) is the absorption coefficient that are determined by consideration of the temperature and excitation photon energy ^3^.

Under steady-state conditions, the decay rate equals the generation rate, leading to

*N* = *g* /($\frac{1}{\tau_{nrad}}$ + $\frac{1}{\tau_{rad}}$), (S3)

($\frac{1}{\tau_{nrad}}$ + $\frac{1}{\tau_{rad}}$)^−1^ represents as the decay time obtained from the experiment. According to the time-resolved PL measurement shown in Fig. S12, the lifetime of *τ*_2_ and *τ*_3_ is used. Thus, we evaluated the exciton density of approximately 9 × 10^11^ cm^−2^ that is comparable to the density of moiré potential at a twist angle of 56.5° ± 0.3°.

**Supplementary Note 3. Calculation of the laser power density**

The spatial beam profile of the continuous-wave laser follows the Gaussian distribution function in the transverse direction to the *z*-axis. The intensity distribution can be given by:

*I*(*r*, *z*) = *I*_z_ exp ($\frac{-2r^{2}}{{W(z)}^{2}})$, (S4)

where *r* (= $\sqrt{x^{2}+y^{2}}$) denotes the radial distance from the *z*-axis, *W*(*z*) represents the radius of the laser profile, and *I*_z_ is the amplitude at *z* = 0 in. the focused plane. The average excitation power density at a given pillar size *D* can be calculated as follows:

*P*_avr_ = $\frac{\oint_{0}^{D} I \left( r \right)dr}{S (r)}$ , (S5)

where *S*(*r*) is the areal size with radius *r*, denoted as *πr*^2^.

**Supplementary Figure 2: PL spectra in the nanofabricated-MoSe_2_/WSe_2_ heterobilayer with various pillar sizes measured at different positions at 4 K and weak excitation conditions of 2 W/cm^2^ (a)** Normalized PL spectra of moiré excitons in the MoSe_2_/WSe_2_ heterobilayer without and with nanofabrication for various pillar sizes. **(b)** Calculated integrated and averaged laser intensity as a function of square of pillar sizes, assuming that the diameter of the Gaussian profile of the focused laser spot is 1.5 μm. **(c)** Integrated PL intensity as a function of squared diameter, denoted by solid circles. Blue squares indicate the calibrated results.

**Supplementary Figure 3: SEM images of the nanofabricated-MoSe_2_/WSe_2_ heterobilayer with various pillar sizes.** The etched regions of outer circles are designed to have a diameter of 2 μm, which indicated by white circles in the figures. The shape and size of the inner pillars are determined by the designed pattern using electron beam lithography. The designed diameters of pillars are shown in the figures.

**Supplementary Figure 4: PL intensities as a function of excitation power densities.** PL intensities of peaks at 1.380 and 1.382 eV as a function of excitation power density with logarithmic and linear scales, with the *x*-axis representing the logarithmic scale **(a)** and linear scale **(b)**.

**Supplementary Figure 5: Temperature dependence of PL spectra of a single moiré exciton measured at a power density of 2 W/cm^2^ (a)** Contour plot of the PL spectrum from 4 to 45 K. **(b)** Normalized PL spectra measured at various temperatures. **(c)** Peak position and spectral linewidth defined by full width at half maximum (FWHM) as a function of temperature.

**Supplementary Figure 6: Statistical data of PL spectra in the heterobilayers. (a)–(e)** PL spectra of the nanopillars with a diameter of 50 nm measured at various nanopillar positions. **(f)** Optical image of the nanofabricated heterobilayer. Red squares indicate the measured positions in the spectra.

**Supplementary Figure 7: Heterobilayer sample with reversed stacking order of WSe_2_ and MoSe_2_. (a)** Optical image of WSe_2_/MoSe_2_ after the same RIE fabrication process. **(b)** Low-temperature PL spectra for various excitation power densities. **(c)** Peak intensities as a function of excitation power density. **(d)** Polar plot of SHG intensities of the heterobilayer in the monolayer WSe_2_ (green), MoSe_2_ (black), and heterobilayer (red) regions.

**Supplementary Figure 8: Circularly polarized PL spectra for various excitation power. (a)** Co- and cross-polarized PL spectra obtained by excitation using a circular polarized laser of different power. **(b)** Degree of circular polarization (DOCP) as a function of excitation power.

**Supplementary Figure 9: Result of linear polarization. (a)** Contour plot of the PL spectrum at various detection angles. **(b)** PL intensity of the emission signal as a function of the linear detection angle.

**Supplementary Figure 10: Time-trace of PL spectra for a single moiré exciton measured at 4 K and excitation power density of 34 W/cm^2^. (a)** PL spectra of a single moiré exciton in the heterobilayer. Each spectrum was measured for an accumulation time of 30 s. The time-trace PL spectra show spectral wandering. **(b)** Time-trace of the spectral peak positions gathered from the spectra. The color bars represent the energy windows with a constant width, in which the frequencies of the peak positions are counted. The distribution range of the peak position is 0.6 meV and ranges from 1.3297 to 1.3303 eV. **(c)** Frequency of the peak positions counted in each energy window, wherein the distribution of frequencies in the energy peak positions can be fitted with a Gaussian function, as indicated by red solid curve. The center and width of the Gaussian distribution are 0.2 meV and 1.3300 eV, respectively.

**Supplementary Figure 11: Optical setup for the low-temperature PL measurement and coherence measurement.** In the measurement of the first-order correlation function, a Michelson interferometer is inserted before the detector.

**Supplementary Figure 12: PL spectra measured at 4 K for various power densities (a)** Power dependence of PL spectra at 4 K in the heterobilayer. **(b)** Integrated PL intensity and linewidth represented by FWHM, which are indicated by black solid squares and sky-blue circles, as a function of the excitation power density. Solid line exhibits the guide for the linear power dependence.

**Supplementary Figure 13: Temperature dependence of PL decay profiles of moiré exciton at an excitation power density of 28 W/cm^2^ (a)** Time evolution of the PL intensity for moiré exciton measured from 4 to 14 K. A supercontinuum laser with a repetition rate of 1 MHz filtered by 1.72-eV band pass filter was used. The multi-exponential functions are used for data fitting, as presented by black solid curves. **(b)** PL lifetimes obtained from the fitted results as a function of temperature.

**Supplementary Figure 14: Interferogram of a single moiré exciton measured at 4 K under various excitation power densities.** The fringe represents the maximum and minimum PL intensities at various delay times. Decays of fringe visibility obtained from the maximum and minimum intensities as a function of delay time are plotted in Fig. 3i.

**Supplementary Figure 15: Interferogram of a single moiré exciton measured at an excitation power density of 40 W/cm^2^ at various temperatures.** Decays of fringe visibility obtained from the maximum and minimum intensities as a function of delay time are plotted in Fig. 3g.

 **Supplementary Figure 16: Time-trace of PL spectra of a single moiré exciton measured at 4 K and an excitation power density of 3.2 W/cm^2^. (a)** Time-trace of the PL spectra of a single moiré exciton. Each spectrum is measured for an accumulation time of 30 s. The time-trace PL spectra present smaller spectral wandering than in Supplementary Fig. 10. **(b)** Time-trace of the spectral peak positions recorded from the spectra. The results show that the distribution range of peak position is 0.2 meV from 1.3271 to 1.3273 eV, which is smaller than the range used from previous results. **(c)** Frequency of peak positions obtained at each energy window. The distribution of frequencies in the energy peak positions can be fitted with a Gaussian function, as indicated by red solid curve. The center and width of the Gaussian distribution are 0.07 meV and 1.3272 eV, respectively.

**Reference**

1. Pasqual Rivera et al. Valley-polarized exciton dynamics in a 2D semiconductor heterostructure. *Science* **351**,688-691 (2016).
2. Fang, H., Lin, Q., Zhang, Y. et al. Localization and interaction of interlayer excitons in MoSe_2_/WSe_2_ heterobilayers. *Nat. Commun.* **14**, 6910 (2023).
3. Liu, HL., Yang, T., Chen, JH. et al. Temperature-dependent optical constants of monolayer MoS_2_, MoSe_2_, WS_2_ and WSe_2_: spectroscopic ellipsometry and first-principles calculations. *Sci. Rep.* **10**, 15282 (2020).
